# Polysaccharide utilization loci of North Sea *Flavobacteriia* as basis for using SusC/D-protein expression for predicting major phytoplankton glycans

**DOI:** 10.1038/s41396-018-0242-6

**Published:** 2018-08-15

**Authors:** Lennart Kappelmann, Karen Krüger, Jan-Hendrik Hehemann, Jens Harder, Stephanie Markert, Frank Unfried, Dörte Becher, Nicole Shapiro, Thomas Schweder, Rudolf I. Amann, Hanno Teeling

**Affiliations:** 1 Max Planck Institute for Marine Microbiology, Bremen, Germany; 2 Zentrum für Marine Umweltwissenschaften, Bremen, Germany; 3 Pharmaceutical Biotechnology, University Greifswald, Greifswald, Germany; 4 Institute of Marine Biotechnology, Greifswald, Germany; 5 Institute for Microbiology, University Greifswald, Greifswald, Germany; 6 DOE Joint Genome Institute, Walnut Creek, CA USA

**Keywords:** Marine microbiology, Water microbiology, Microbial ecology

## Abstract

Marine algae convert a substantial fraction of fixed carbon dioxide into various polysaccharides. *Flavobacteriia* that are specialized on algal polysaccharide degradation feature genomic clusters termed polysaccharide utilization loci (PULs). As knowledge on extant PUL diversity is sparse, we sequenced the genomes of 53 North Sea *Flavobacteriia* and obtained 400 PULs. Bioinformatic PUL annotations suggest usage of a large array of polysaccharides, including laminarin, α-glucans, and alginate as well as mannose-, fucose-, and xylose-rich substrates. Many of the PULs exhibit new genetic architectures and suggest substrates rarely described for marine environments. The isolates’ PUL repertoires often differed considerably within genera, corroborating ecological niche-associated glycan partitioning. Polysaccharide uptake in *Flavobacteriia* is mediated by SusCD-like transporter complexes. Respective protein trees revealed clustering according to polysaccharide specificities predicted by PUL annotations. Using the trees, we analyzed expression of SusC/D homologs in multiyear phytoplankton bloom-associated metaproteomes and found indications for profound changes in microbial utilization of laminarin, α-glucans, β-mannan, and sulfated xylan. We hence suggest the suitability of SusC/D-like transporter protein expression within heterotrophic bacteria as a proxy for the temporal utilization of discrete polysaccharides.

## Introduction

Half of global net primary production is oceanic and carried out mostly by small, unicellular phytoplankton such as diatoms [1]. Polysaccharides account for up to 50% of algal biomass [2] and can be found as intracellular energy storage compounds, as structural components of their cell walls [3], or as secreted extracellular transparent exopolymeric substances [4]. They can be composed of different cyclic sugar monomers linked by either α- or β-glycosidic bonds at different positions and can be substituted by different moieties (e.g., sulfate, methyl, or acetyl groups), making them the most structurally diverse macromolecules on Earth [5].

Many members of the bacterial phylum *Bacteroidetes*, including marine representatives of the class *Flavobacteriia*, are specialized on polysaccharide degradation. They feature distinct polysaccharide utilization loci (PULs, [6]), i.e., operons or regulons that encode the protein machinery for binding, degradation and uptake of a type or class of polysaccharides. Polysaccharides are initially bound by outer membrane proteins and cleaved by endo-active enzymes into oligosaccharides suitable for transport through the outer membrane. Oligosaccharides are bound at the interface of SusCD complexes. SusD-like proteins are extracellular lipoproteins and SusC-like proteins constitute integral membrane beta-barrels termed TonB-dependent transporters (TBDTs). Glenwright et al. [7] showed that these two proteins form a ‘pedal bin’ complex in *Bacteroides thetaiotaomicron*, with SusD acting as a lid on top of the SusC-like TBDT. Upon binding of a ligand, the SusD lid closes and conformational changes lead to substrate release into the periplasm. Here, further saccharification to sugar monomers takes place that are taken up into the cytoplasm via dedicated transporters.

Besides the characteristic *susCD*-like gene pair, *Bacteroidetes* PULs contain various substrate-specific carbohydrate-active enzymes (CAZymes), such as glycoside hydrolases (GHs), polysaccharide lyases (PLs), carbohydrate esterases (CEs), carbohydrate-binding modules (CBMs), and proteins with auxiliary functions. PULs of human gut *Bacteroidetes* and their capacity to degrade various land plant polysaccharides have been thoroughly investigated (e.g., ref. [8]), but knowledge on marine polysaccharide degradation is sparse. Many polysaccharides in marine algae differ from those in land plants. Green macroalgae contain ulvans, red macroalgae contain agars, carrageenans and porphyrans, brown algae contain alginates, fucans and laminarin, and diatom microalgae contain chrysolaminarin and sulfated mannans, all of which are presumably absent in land plants [9]. Likewise, many algae feature anionic, sulfated polysaccharides that require sulfatases for degradation.

A systematic inventory of the structural diversity of algal polysaccharides has not yet been achieved. We do not have a good understanding of the associated diversity of PULs in marine *Bacteroidetes*. Also only few PULs have so far been linked to their polysaccharide substrate. Examples include an agar/porphyran-specific PUL [10] that human gut *Bacteroidetes* acquired from marine counterparts [11], an alginate-specific PUL in *Zobellia galactanivorans* DsiJ^T^ [12], alginate- and laminarin-specific PULs in *Gramella forsetii* KT0803 [13], a similar laminarin-specific PUL in *Polaribacter* sp. Hel1_33_49 [14], and a complex carrageenan degradation regulon in *Z. galactanivorans* DsiJ^T^ [15]. Few overarching comparative genomic studies exist [14, 16], focusing largely on overall CAZyme repertoires.

Pioneering studies on structural elucidation of polysaccharides from microalgae were performed [17, 18], but precise microalgal polysaccharide structures remain mostly unresolved (for review, see ref. [4]), because they require sophisticated methods [19]. PUL analysis of heterotrophic bacteria co-occurring with phytoplankton could serve as an alternative starting point to advance insight into the structures of marine polysaccharides and to understand their microbial decomposition.

Here we present a comparative analysis of PULs from 53 newly sequenced *Flavobacteriia* isolated from the German Bight, comprising a total of 400 manually determined PULs. Based on these data we investigated whether SusC- and SusD-like sequences can be linked to distinct predicted polysaccharides. Using environmental metaproteome data we show how SusC/D homolog expression may be used to assess the presence of marine polysaccharides during North Sea spring blooms.

## Materials and methods

### Isolation and sequencing of North Sea *Flavobacteriia*

*Flavobacteriia* were sampled at the North Sea Islands Helgoland and Sylt as described previously ([20, 21], Supplementary Table S1). Also included were the previously sequenced *Gramella forsetii* KT0803 [22], *Polaribacter* spp. Hel1_33_49 and Hel1_85 [14], and the *Formosa* spp. Hel1_33_131 and Hel3_A1_48. The remaining 48 genomes were sequenced at the Department of Energy Joint Genome Institute (DOE-JGI, Walnut Creek, CA, USA) in the framework of the Community Sequencing Project No. 998 COGITO (Coastal Microbe Genomic and Taxonomic Observatory). Forty genomes were sequenced using the PacBio RSII platform exclusively, whereas eight isolates were sequenced using a combination of Illumina HiSeq 2000/2500 and PacBio RSII. All these genomes are GOLD certified at level 3 (improved high-quality draft) and are publicly available at the DOE-JGI Genomes OnLine Database (GOLD, [23]) under the Study ID Gs0000079.

### Gene and PUL annotation

Initial annotations of the genomes of *Polaribacter* spp. Hel1_44_49 and Hel1_85 and *Formosa* spp. Hel1_33_131 and Hel3_A1_48 were performed using the RAST annotation system [24]. All other genomes were annotated using the DOE-JGI Microbial Annotation Pipeline (MGAP, [25]). These annotations were subsequently imported into a GenDB v2.2 annotation system [26] for refinement and additional annotations based on similarity searches against multiple databases as described previously [27].

SusC- and SusD-like proteins were annotated by the DOE-JGI MGAP, which uses the TIGRfam model TIGR04056 to detect SusC-like proteins and the Pfam models 12741, 12771, and 14322 to detect SusD-like proteins. CAZymes were annotated based on HMMer searches against the Pfam v25 [28] and dbCAN 3.0 [29] databases and BLASTp searches [30] against the CAZy database [31]. CAZymes were annotated only as such when at least two of the database searches were positive based on family-specific cutoff criteria that were described previously [32]. Selected sulfatases were annotated using the SulfAtlas database v1.0 [33]. Peptidases were annotated using BLASTp searches against the MEROPS 9.13 database [34] using the default settings of E ≤ 10^−4^.

PULs were manually detected based on the presence of CAZyme clusters, which in most cases also featured co-occurring *susCD*-like gene pairs as previously suggested [6]. In some cases, the sequence similarity of a TBDT was too low to be considered SusC-like, no SusD homolog was present or the entire *susCD*-like gene tandem was missing. These operons were still counted as PULs and are regarded as incomplete subtypes [35].

### Gene expression analyses of *Flavobacteriia*-rich North Sea bacterioplankton using metaproteomics

During spring phytoplankton blooms of 2009 to 2012, 14 surface seawater biomass samples were collected at the long-term ecological research station ‘Kabeltonne’ (54° 11.3’ N, 7° 54.0’ E) off the German North Sea island Helgoland as previously described in detail [32, 36]. Biomass was collected on 0.2 µm pore sized filters after pre-filtration with 10 and 3 µm pore sized filters. Metagenome sequencing was done using the 454 FLX Ti platform for 2009 and the Illumina HiSeq 2000 platform for 2010 to 2012 samples [32].

Corresponding metaproteome analyses were performed from biomass obtained from the same water samples. Protein extraction from 0.2 µm filtered bacterioplankton biomass and separation was carried out as described previously [36] with the modification that gel lanes were cut into 10 equal pieces prior to tryptic digestion (1 µg/ml, Promega, Madison WI, USA) and subsequent mass spectrometric detection in an LTQ Orbitrap Velos mass spectrometer (Thermo Fisher, Bremen, Germany). The mass spectrometry proteomics data have been deposited to the ProteomeXchange Consortium via the PRIDE partner repository [37]; data set identifiers: PXD008238, 10.6019/PXD008238.

Mass spectrometric data were analyzed using Sequest v27r11 (Thermo Fisher Scientific, San Jose, CA, USA). Searches were carried out against a forward-decoy database of all proteins from all metagenome samples combined. This non-redundant database was constructed from all predicted protein-coding genes of all metagenomes (6,194,278 sequences) using the uclust option of USEARCH v6.1.544 [38]; options: cluster_fast; nucleotide identity 0.99; maxhits 5; maxrejects 30) and contained 3,212,324 sequences. Common laboratory contaminants were included in all databases. Technical duplicates of each sample were searched together (including all 20 subsamples) to obtain averaged spectral counts. Validation of protein- and peptide identifications was performed with Scaffold v4 (Proteome Software Inc, Portland, OR, USA) using the parameters previously described [36], and normalized spectral abundance factors (%NSAF) were calculated [39] to allow for semi-quantitative analyses (Supplementary Table S2). The NSAF quantitation measure is commonly used in non-gel-based label-free shotgun proteomics. In brief, a %NSAF of 1 corresponds to 1% of all mass-adjusted spectral count data in a given proteomic experiment.

### SusC/D homolog tree reconstruction

We constructed trees from SusC- and SusD-like protein sequences of the isolates’ PULs (SusC: 369; SusD: 361). Sequences were aligned using MAFFT v7.017 [40] using the G-INS-i algorithm and BLOSUM62 matrix with default gap open penalty (1.53) and offset (0.123) values. Maximum-likelihood trees were constructed using FastTree 2.1.5 [41] with default settings.

## Results

### High genomic and phylogenetic diversity in isolated marine *Flavobacteriia*

**Fig. 1:**
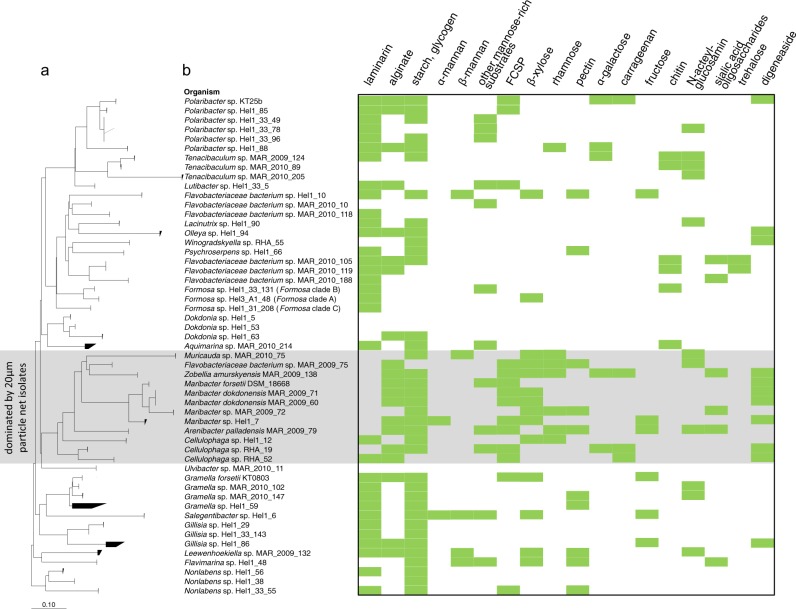
**a** Maximum-likelihood tree of 53 North Sea *Flavobacteriia* isolates based on full-length 16 S rRNA gene sequences. Scale bar: 10 nucleotide substitutions per 100 nucleotides. **b** Predicted degradation capacities of polysaccharide classes based on PUL-associated CAZyme annotations

The 53 flavobacterial isolates cover a broad range of the *Flavobacteriia* class within the phylogenetic tree based on full-length 16S rRNA genes (Fig. 1). The strains fall into several clusters that can be linked to characteristic genomic features (Supplementary Table S1). Genome sizes ranged from 2.02 Mbp (*Formosa* sp. Hel3_A1_48) to 5.98 Mbp (*Aquimarina* sp. MAR_2010_214), with an average of 3.83 Mbp. One of the clusters was dominated by isolates obtained from the retentates of seawater filtered through 20 µm particle nets (8 out of 12; Fig. 1; Supplementary Table S1). These species feature mostly larger genomes (average 4.5 Mbp) and are likely associated with microalgae. Forty-seven of the 53 strains have two to four 16S rRNA operons, with the notable exception of the three *Tenacibaculum* strains possessing six (strains MAR_2009_124 and MAR_2010_205) and seven (strain MAR_2010_89), respectively.

The capacity of the isolates to degrade polysaccharides varied widely as indicated by the number of degradative CAZymes per Mbp and predicted PULs per genome. On average, we identified 7.5 PULs per genome and 55 degradative CAZymes (Supplementary Table S1). Strains of the putative microalgae-associated cluster differed with on average 83.3 degradative CAZymes, almost twice as many PULs per genome (14.2) and many sulfatase genes, indicating an extended capacity for the degradation of sulfated polysaccharides (average of 28.2 sulfatases, with a maximum of 95 sulfatases in *Zobellia amurskyensis* MAR_2009_138). The other strains had an average of 46.8 degradative CAZymes and 5.5 PULs. Eleven isolates possessed less than three PULs, contained few (≤ 3) or no sulfatases and were exclusively isolated from surface seawater or pore water. They likely target rather simple, non-sulfated polysaccharides and peptides. This strategy is emphasized by their high peptidase:CAZyme ratio of 1.81, compared with an average ratio of 0.95 for isolates with > 10 PULs. Still it is noteworthy that numbers of PULs and degradative CAZymes varied considerably, even within isolates of the same genus.

### Putative substrate specificities

The 53 genomes revealed a wide range of as yet undescribed PULs. In total, 400 PULs were annotated, 259 of which could be linked to either dedicated polysaccharides or polysaccharide classes by in-depth annotations (Supplementary Table S3).

### Known and putative new laminarin PULs

**Fig. 2:**
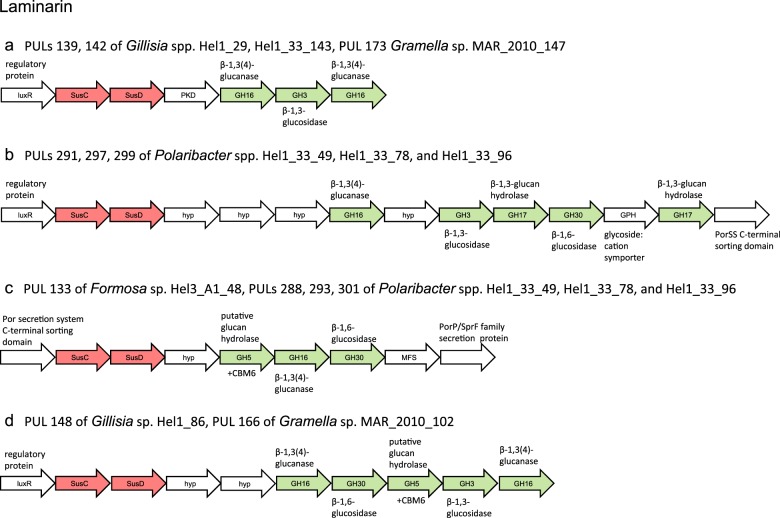
Conserved PULs known (**a**, **b)** and predicted (**c**, **d)** to target laminarin

Laminarins are β-1,3-linked glucans that are abundant as they act as storage compounds in brown algae and diatoms (both *Stramenopiles*). Forty-six PULs (~ 12%) were predicted to be involved in laminarin degradation, featuring four variants (Fig. 2): Variant A is a highly conserved, short PUL containing one predicted GH3 β-1,3-glucosidase framed by two predicted GH16 β-1,3(4)-glucanases (Fig. 2a). This arrangement was first described in *Gramella forsetii* KT0803 and shown to be upregulated by laminarin [13]. Two distinct GH16 laminarinases have been studied in *Z. galactanivorans* DsiJ^T^, which are endo-active [42, 43]. For this species, it has been speculated that a cell surface-associated GH16 glucanase cleaves branched laminarin polysaccharides into oligosaccharides [43], which can be transported through the SusC-like TBDT into the periplasm. Here, the GH3 β-1,3-glucosidase may further cleave off glucose units [44], which are imported into the cytoplasm.

Variant B is a larger, more variable PUL (Fig. 2b). It shares homology with a PUL in *Polaribacter* sp. Hel1_33_49 that can be induced by laminarin [14]. This PUL additionally features a predicted GH30 exo-β-1,6-glucanase and at least two GH17 β-1,3-glucan hydrolases with predicted endo- and exo-activities, respectively. The GH30 exo-β-1,6-glucanase removes β-1,6-glucose side chains from laminarin [45]. Although GH16 enzymes can hydrolyze both β-1,3- and β-1,4-linked glucans, GH17 glucan hydrolases are highly specific to undecorated β-1,3 glucans and can have endo- [46] and exo-activity [47]. The β-1,3-glucan endohydrolase thus likely cleaves laminarin into oligosaccharides, which may be further degraded into glucose by the β-1,3-glucan exohydrolase.

Variants C and D PULs are likewise predicted to be capable of laminarin degradation based on gene content but have not been described before (Fig. 2c and d). They feature an additional putative GH5 glucan hydrolase with a carbohydrate-binding domain that binds β-1,3- and β-1,4-glucans (CBM6c, [48]). They furthermore contain GH16 and GH30 family enzymes as described in variant B, but no GH17 enzymes.

In total, 62% (33/53) of all isolates and 78% (25/32) of surface water isolates contained at least one laminarin PUL. Variant A occurred 21 times, B 17 times, C five times, and D two times (Supplementary Table S3). Eight isolates possessed two laminarin PULs (*Flavobacteriaceae bacterium* spp. MAR_2010_105 and MAR_2010_119, *Gramella* sp. MAR_2010_102, *Polaribacter* spp. Hel1_33_49, 78, 96 and Hel1_88 and *Psychroserpens* sp. Hel1_66) and three isolates contained three (*Formosa* spp. Hel3_A1_48 and Hel1_33_131, *Flavobacteriaceae bacterium* sp. Hel1_10). In contrast, laminarin PULs were far less prevalent in isolates obtained from the >20 µm retentate (2/12). Laminarins are composed of a β-1,3-glucan backbone ramified by β-1,6 and, less frequently, β-1,2-linked glucose side chains [49]. The backbone length and ramification degree varies in different species. Laminarin of brown algae is capped at the reducing end by a 1-linked d-mannitol [50]. Only three isolates with laminarin PULs, namely the *Polaribacter* spp. Hel1_85 and KT25b and *Gramella* sp. MAR_2010_102, also possessed an annotated mannitol-2-dehydrogenase. It is possible that this enables utilization of brown algal laminarin. However, free mannitol is a more likely substrate. Growth on free mannitol has for example been demonstrated in the marine flavobacterium *Z. galactanivorans* [51]. Studies on *Ectocarpus siliculosus* have shown that brown algae can store substantial amounts of free mannitol as compatible osmolyte [52]. Furthermore it has recently been shown that free mannitol is likewise frequently found in various planktonic microalgae [53]. Interestingly, diatoms seem to have lost their ability to synthesize mannitol, although exceptions exist [53]. The fact that phytoplankton blooms in the southern North Sea are usually diatom-dominated would hence explain, why mannitol-2-dehydrogenase genes were rarely found in our isolates. Consequently, the majority of isolates with laminarin PULs seem to only target diatom-type non-mannitol-capped chrysolaminarins, indicating that these are the major available laminarins in the southern North Sea.

### α-1,4-glucan (starch, glycogen)

**Fig. 3:**
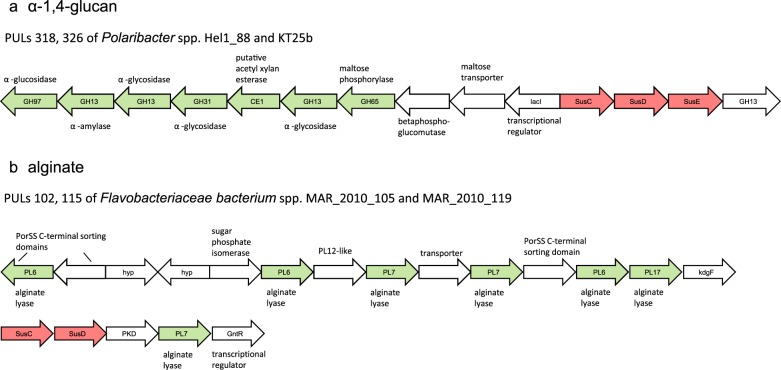
PULs predicted to target **a** α-1,4-glucans and **b** alginate

PULs predicted to target α-1,4-glucans, such as starch, glycogen, and amylose were also highly abundant (43/400 PULs, 37/53 isolates; Supplementary Table S3). Respective PULs often featured a *susCDE*-like gene triplet, at least one predicted GH13 α-glycosidase and frequently GH65 phosphorylases as well as GH31 hydrolases acting on α-glucosidic linkages (Fig. 3a). In some cases, these PULs also encoded a GH97 family glycoside hydrolase, known to hydrolyze diverse α-1,2-, α-1,3-, α-1,4-, and α-1,6-linked glycosidic bonds [54, 55]. A similar PUL was first described for *Gramella forsetii* KT0803 and found to be upregulated in response to glucose-polymer substrates [13]. The PUL depicted in Fig. 3a likely facilitates utilization of α-1,4-glucans featuring α-1,6-branches, such as the starch molecule amylopectin or potentially bacterial glycogen. Contrastingly, some isolates featured reduced versions of this PUL without any annotated *susE*- or *susF*-like gene and only one GH13 and GH65 gene, respectively (e.g., all *Maribacter* isolates). These isolates may only target simple non-branched α-1,4-glucans such as maltodextrin or amylose. Recent investigations have shown that *B. thetaiotaomicron* SusE is an immobile outer membrane protein that can modify the preferred sizes of maltooligosaccharides for uptake [56, 57]. Hence SusE homologs, whereas not essential, might be generally involved in fine-tuning the size selection of glycan uptake.

### Alginate

Twenty isolates featured in total 27 alginate-specific PULs (Fig. 1). Alginate consists of blocks of β-D-mannuronic acid and α-l-guluronic acid, forming a linear β-1,4-linked chain [58]. Corresponding PULs encode family PL6, 7, and 17 alginate lyases (Fig. 3b). Six of the alginate PULs also contained genes with sequence similarities to the sparsely investigated PL12 family. Known PL12 enzymes cleave heparin—a polymer of β-1,4-linked uronic acids and glucosamine that is often highly sulfated [59]. Heparin and alginate hence are both linear, β-1,4-linked C5-uronans. However, sulfated alginates analogous to heparin, whereas being artificially synthesized for biotechnological uses [60–62], have not been reported in nature. The latter is in line with a lack of sulfatases in the alginate PULs. Therefore, the PL12 family enzymes encoded in the alginate PULs likely represent novel types of alginate lyases. No PL15 and only one potential PL14 family alginate lyase (*Lutibacter* sp. Hel1_33_5, not PUL-associated) were annotated. The putative microalgae-associated cluster had a higher prevalence (8/12) of alginate PULs as compared with the other isolates (12/41) (Fig. 1).

### Mannose-rich substrates

Seventeen isolates harbor PULs rich in mannose-targeting CAZyme genes, e.g., from families GH26, 38, 76, 92, 125, and 130 (Fig. 1, Supplementary Table S5). These PULs share little conservation in terms of gene arrangements, and only few contain a GH76 endo-α-1,6-mannanase (2/17) or a GH26 family endo-β-1,4-mannanase (5/17), indicative for either a linear α-1,6- or β-1,4-mannan backbone. Instead, GH92 and GH130 family genes are particularly prevalent. The GH92 family comprises solely exo-acting α-mannase genes. PULs rich in GH92 genes thus might target α-mannose-rich *N*-glycosylated glycoproteins that occur widespread in eukaryotes, including algae. GH130 enzymes comprise phosphorylases and glycoside hydrolases that act on β-mannosides and are known to partake in the degradation of β-mannans [63].

**Fig. 4:**
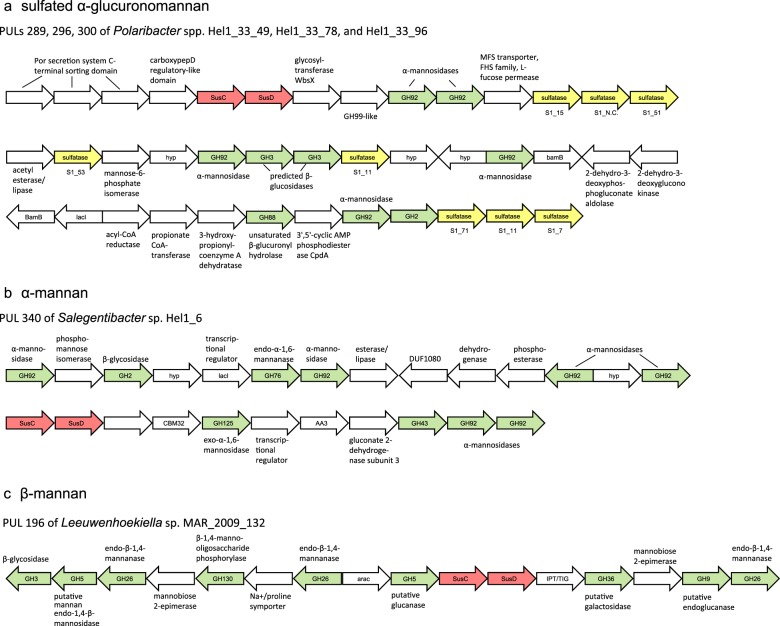
Selected PULs predicted to target mannose-rich substrates. Possible targets are **a** a sulfated α-mannan, **b** a non-sulfated α-mannan, and **c** a non-sulfated β-mannan. For sulfatases, families and sub-families are indicated below the genes

The alpha-mannosidase-encoding PULs can be divided into two subtypes: (A) PULs containing multiple GH92 (α-1,2/3/4/6) genes that are often also rich in sulfatase genes (Fig. 4a; e.g., Supplementary Table S3: PULs 289, 296, 300 of *Polaribacter* spp. Hel1_33_49/78/96; PUL126 of *Formosa* sp. Hel1_33_131), and (B) PULs with α-mannan-targeting CAZymes of diverse additional families, such as GH76 endo-α-1,6-mannanases, GH125 exo-α-1,6-mannosidases or GH38 α-mannosidases (α-1,2/3/6). These type (B) PULs are notably devoid of sulfatase-coding genes, indicating a non-sulfated substrate (Fig. 4b). A PUL with a similar CAZyme repertoire in *B. thetaiotaomicron* facilitates utilization of yeast cell wall α-mannan [64]. Type (A) sulfatase- and GH92-rich PULs have been observed previously in *Polaribacter*-affiliated North Atlantic fosmids [65] and *Polaribacter* sp. Hel1_33_49 [14] and therefore seem to be widespread. In our case the PUL contains additional GH2, 3 and 88 family enzymes (Fig. 4a). Whereas GH families 2 and 3 are functionally diverse, GH88 enzymes are unsaturated β-glucuronyl hydrolases. This functional combination of CAZymes suggests degradation of α-glucomannans such as glucuronomannan, a polysaccharide that has been reported for diatoms [17, 19, 49] and brown algae [66] and thus should be abundant in the southern North Sea. Finally, co-located peptidases and a gene distantly related to GH99, a family that is reported to contain glycoprotein endo-α-mannosidases, indicate that this hypothesized glucuronomannan substrate might be a glycoprotein. However, functional studies are required to support this hypothesis. Le Costaouëc and colleagues [19] recently revealed the main cell wall polysaccharide of the diatom *Phaeodactylum tricornutum* and possibly many other diatoms [67] to be a ‘linear poly-α-1–3-mannan decorated with sulfate ester groups and β-d-glucuronic residues’.

The β-mannan PULs (Fig. 4c) all contained GH130 β-1,2(4)-mannooligosaccharide phosphorylases and GH26 CAZymes which are primarily composed of predicted endo-β-1,4-mannanases (Supplementary Table S3: PUL 6 of *Flavobacteriaceae bacterium* sp. Hel1_10; PUL 196 of *Leeuwenhoekiella* sp. MAR_2009_132; PUL 211 of *Flavimarina* sp. Hel1_48; PUL266 of *Muricauda* sp. MAR_2010_75; PUL 342 of *Salegentibacter* sp. Hel1_6). Beta-mannans have been reported in the red macroalga *Porphyra umbilicalis* and in various species of the green macroalga *Codium*. Moreira and Filho [68] proposed that ‘in some algae species, linear (beta-) mannan seems to replace cellulose as the main cell wall glycan’.

### Fucose-containing sulfated polysaccharides (FCSP)

**Fig. 5:**
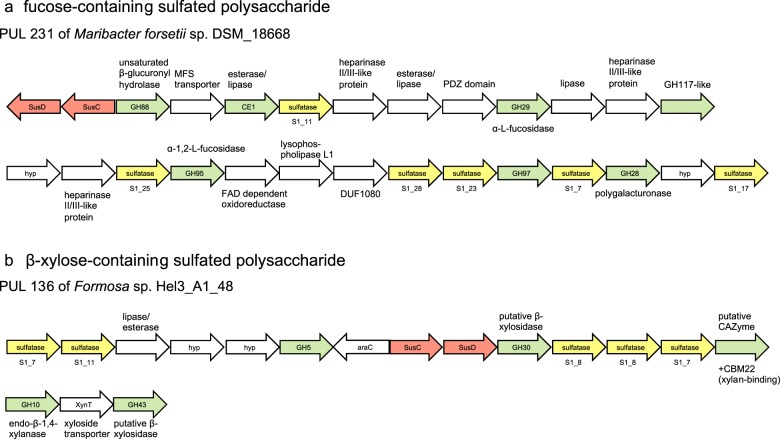
PULs predicted to target sulfated substrates rich in **a** fucose (FCSP) and **b** xylose. For sulfatases, families, and sub-families are indicated below the genes

Twenty PULs in 14 isolates suggest that FCSPs are also common substrates to marine *Flavobacteriia*. A prominent substrate of this group is fucoidan, a highly diverse polysaccharide prominent in brown algae. It contains l-fucose and sulfate ester groups owing to its backbone of α-1,3 or alternating α-1,3/1,4-linked l-fucopyranosyl residues [69]. This backbone has side chains containing diverse other monosaccharides, uronic acids, acetyl groups, and proteins [70]. In accordance with the structural complexity of FCSPs, PULs display equally complex gene repertoires, averaging 38 genes per PUL. A relatively short PUL is exemplarily shown in Fig. 5a. Characteristic CAZymes of predicted FCSP PULs in the isolated *Flavobacteriia* were GH29 and GH95 family α-l-fucosidases or potentially α-l-galactosidases. Other regularly co-occurring CAZymes included GH117 family enzymes (Supplementary Table S5), β-xylosidases mostly of the family GH43, but also GH30, 39, and 120, and diverse α- and β-glucosidases of the families GH2, 3, 31, and 97. Sulfated FCSPs such as xylofucoglucans or -glucuronans have been reported for brown algal hemicelluloses [3, 9] and might also occur in diatoms [49].

### β-xylose-containing substrates

Twenty-two PULs predicted to target β-xylose-rich substrates were found in 14 isolates (Fig. 1, Supplementary Table S3). Likely substrates are heterogeneous β-xylans such as arabinoxylans, glucuronoxylans, and sulfated xyloglucans. These PULs encode GH10 and GH43 enzymes targeting xylans, as well as GH30, 115, and 67 enzymes. GH10 enzymes are endo-β-1,4-xylanases capable of cleaving large β-1,4-xylan backbones into oligosaccharides. GH30 and GH43 enzymes have broader degradation capacities, and while both families contain β-xylosidases, they were also reported to target mixed xylose-containing substrates such as arabinoxylan and α-l-arabinofuranosides (GH43) or even completely different substrates such as β-glucosylceramidase or β-1,6-glucanase (GH30). GH67 and 115 can cleave glucuronic acid side chains from native xylans and are present in four PULs that might target glucuronoxylans (PULs 243, 256 of *Maribacter* spp. Hel1_7, MAR_2009_72; PUL 269 of *Muricauda* sp. MAR_2010_75; PUL 338 of *Salegentibacter* sp. Hel1_6; Supplementary Table S3). Two PULs were predicted to target arabinoxylans through a GH51 α-l-arabinofuranosidase (PUL 155 of *Gramella forsetii* KT0803; PUL 199 of *Flavimarina* sp. Hel1_48; Supplementary Table S3). Four PULs predicted to target β-xylose-rich substrates encode sulfatases (PUL136 of *Formosa* sp. Hel3_A1_48, Fig. 5b; PULs 363, 364, 366 of *Zobellia amurskyensis* MAR_2009_138; Supplementary Table S3). Marine xylans have been reported as hemicellulose components in green [71], red and brown macroalgae [3, 72], and as cell wall components in some diatoms [73, 74].

### Further substrates

Further possible substrates comprised sulfated α-rhamnose- and α-galactose-containing substrates, pectin, arabinan, trehalose-like α-1,1-glucans, *N*-acetylglucosamine and its polymer chitin, digeneaside, fructose, and sialic acid-containing polysaccharides. These compounds are discussed in the supplementary text.

### Trees of SusC- and SusD-like proteins reveal substrate-specific clusters

**Fig. 6:**
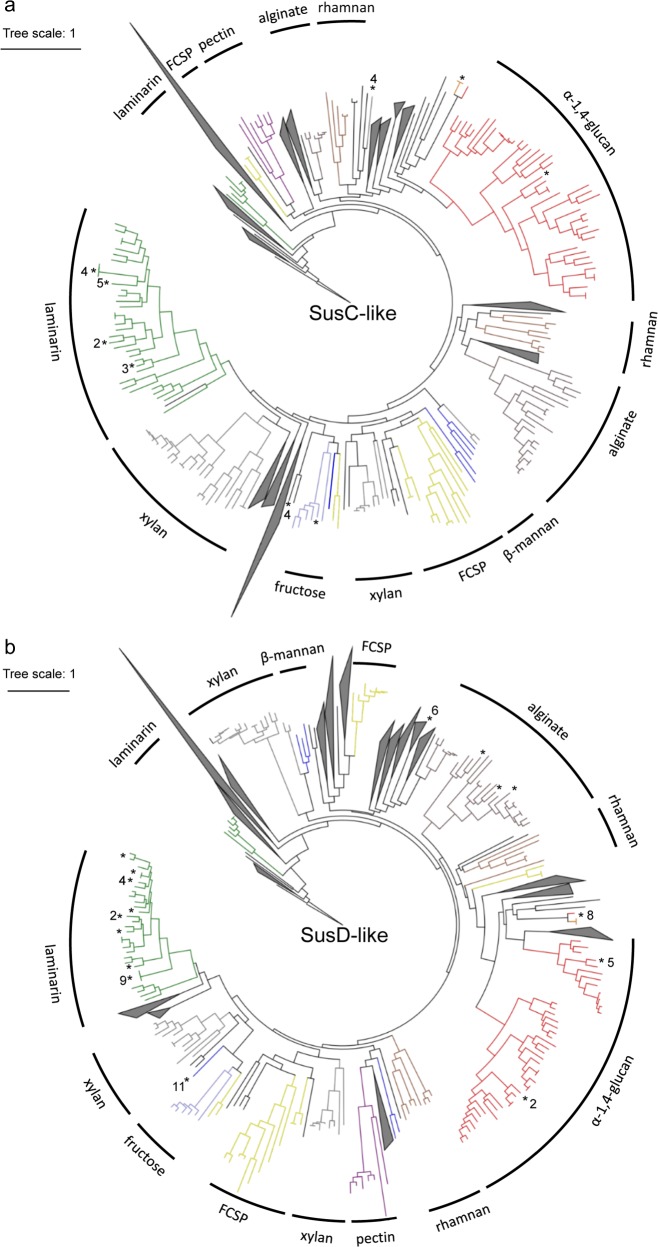
Trees of all PUL-associated SusC- (**a)** and SusD-like (**b)** proteins of the *Flavobacteriia* isolates showing functional, substrate-specific clustering. Protein sequences were aligned using the MAFFT G-INS-i algorithm and trees were calculated using FastTree 2.1.5 approximate-maximum likelihood (SusC-like: 370; SusD-like: 362). Substrate predictions are depicted in colors. Proteins with expressed homologs in North Sea bacterioplankton blooms of more than 40% sequence identity are marked with asterisks (and number of homologs if *x* > 1). Corresponding figures labeled with protein sequence identifiers, originating species and PUL-associated CAZymes are provided as supplementary material

We computed trees for all SusC- and SusD-like protein sequences of the 400 isolate PULs and obtained pronounced clusters for many of the predicted polysaccharide substrates (Fig. 6). For clarity, functionally heterogeneous or undefined clusters are depicted as gray triangles (complete trees: Supplementary files 1, 2). Well-defined clusters in both trees included the structurally simple polysaccharides laminarin, α-1,4-glucans and alginate. For example, SusD-like proteins of laminarin-targeting PULs of *Cellulophaga* sp. RHA_52, *Flavobacteriaceae bacterium* sp. Hel1_10, *Formosa* sp. Hel1_33_131 and *Psychroserpens* sp. Hel1_66 (PULs 58, 71, 128, 331, Supplementary Table S3), exhibited between 64 and 78% identity (Supplementary Table S4), whereas identity to SusD-like sequences from other PULs within the same respective genome was only 10–25% (data not shown).

SusC/D-like proteins from conserved PULs for these structurally simple substrates were more closely related than those from more variable PULs targeting structurally more diverse substrate classes such as FCSPs or xylose-rich substrates (Supplementary Table S4). This is visible in the trees by shorter and longer respective branch lengths (Fig. 6). Some substrates formed multiple clusters, for example xylose-rich substrates. This might indicate either rather different xylose-containing substrates or multiple ways of attack and uptake for a given class of xylose-containing substrate.

The topologies of the SusC- and SusD-like protein trees were notably congruent regarding branching patterns of the identified substrate-specific clusters. Only the pectin cluster was located at a distinctly different position. SusC- and SusD-like proteins from the same PULs exhibited a strong tendency to occur in corresponding substrate-specific clusters in both trees. This applied to > 70% of the SusC and SusD sequences within identified substrate-specific clusters (Supplementary Figures S1A and B).

### SusC/D-like protein expression of bacterioplankton during phytoplankton blooms supports temporal variations of polysaccharide abundances in situ

SusC/D-like proteins range among the highest expressed proteins in bacterioplankton metaproteomes from productive oceans [36, 75, 76]. Likewise, studies on flavobacterial isolates have identified SusC/D-like proteins as the highest expressed proteins within PULs that are furthermore co-regulated with other PUL-encoded proteins including CAZymes [13, 14]. SusC/D expression thus represents a suitable proxy for overall PUL expression

We monitored bacterioplankton spring phytoplankton blooms in the southern North Sea during 2009 with weekly, and in 2010 to 2012 with about monthly sampling [32, 36]. At 14 selected time points we analyzed the free-living 0.2–3 µm bacterioplankton using shotgun metaproteomics (total: 23,917 identified proteins), and detected high numbers of expressed SusC/D-like proteins in metaproteomes across all sampled years (Supplementary Table S2).

**Fig. 7:**
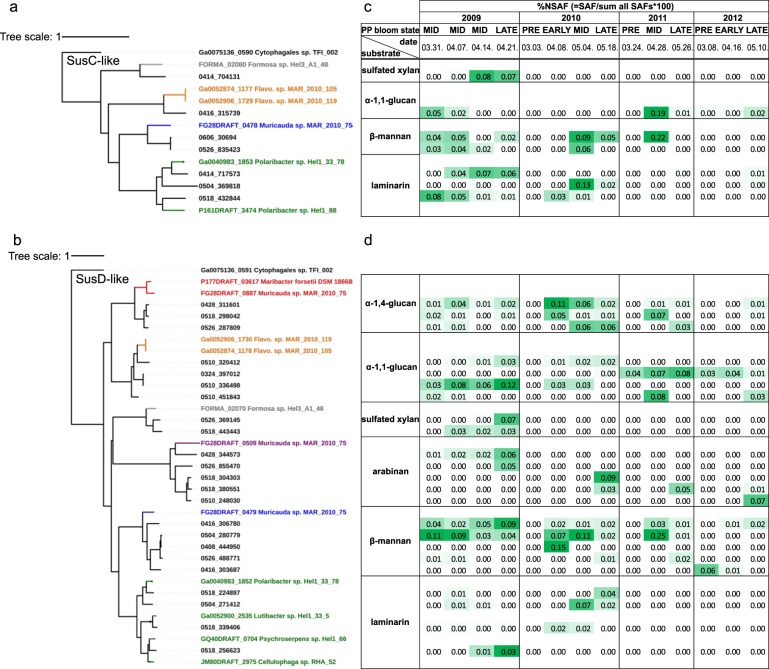
**a**, **b** Trees of expressed SusC and SusD-like proteins identified in 3–0.2 µm bacterioplankton during North Sea spring phytoplankton blooms in 2009–2012 using proteomics. The most closely related SusC/D-like sequences from North Sea *Flavobacteriia* isolates in this study were integrated in the tree. Protein names correspond to sequence identifier and isolate name. Sequences were aligned using the MAFFT G-INS-i algorithm. The tree was calculated using FastTree 2.1.5 approximate-maximum likelihood. **c**, **d** Corresponding expression levels as Normalized Spectral Abundance Factors (%NSAF) for the four consecutive blooms. Metaproteomic samples were classified as pre-, early-, mid-, and late-bloom based on chlorophyll *a* concentrations during the spring phytoplankton blooms. Expression levels are highlighted by green color

To identify potential substrates, we aligned all expressed SusC/D-like sequences (SusC: 390; SusD: 118) to the SusC/D-tree constructed from isolate PULs. Isolate sequences with highest similarities (≥ 40%) to expressed sequences are indicated in Fig. 6. Further semi-quantitative analyses were confined to SusC/D-like proteins where at least one related homolog reached expression levels of ≥ 0.05 %NSAF, i.e. 0.05% of all mass-adjusted spectral counts (see Materials and methods; Fig. 7).

#### Laminarin

Homologs to laminarin-binding SusC-like proteins were detected amidst the 2009 and 2010 phytoplankton blooms, with one homolog reaching a notable maximum of 0.13 %NSAF on May 4th, 2010 (Fig. 7b). Respective SusD-like homologs were detected in the same years and highest expression was observed at the same date in 2010 (0.07 %NSAF, Fig. 7d). Amino-acid identities of expressed SusC homologs and laminarin PUL SusC-like proteins from isolates ranged from 48–68%, and for SusD homologs from 40–78% (Supplementary Table S4). Our data suggest that laminarin occurred at the bloom peaks in 2009 and 2010 and directly thereafter. This is supported by detection of expressed GH3 β-glucosidases and GH16 β-glucanases in 2009 [36] and, to a lesser degree, in 2010 (Supplementary Table S2). Chrysolaminarin is produced by microalgae such as *Thalassiosira nordenskioeldii* diatoms or representatives of *Phaecystis* haptophytes [77, 78], which both were among the dominating microalgae in 2009 and 2010 [32].

#### Alpha-1,4-glucan

Respective SusD-like proteins were most abundantly detected in 2010, peaking on April 8th (0.11 %NSAF, Fig. 7d), but also in 2009 and 2011. Sequence identities to isolate α-1,4-glucan PUL SusD-like proteins ranged from 43 to 46% (Supplementary Table S4). These data indicate that α-1,4-glucans, potentially starch or glycogen, represented a recurring substrate from 2009 to 2011 during early to late phytoplankton bloom stages.

#### Alpha-1,1-glucan

An α-1,1-glucan-binding SusC-like protein potentially targeting a trehalose-like substrate was strongly expressed on April 28th 2011 (0.19 %NSAF; Fig. 7b), but also in all other years except 2010. Its sequence identity to the SusC-like proteins of the trehalose PULs of *Flavobacteriaceae bacterium* spp. MAR_2010_105 and MAR_2010_119 was 43% (Supplementary Figure S2J, PUL103 and PUL116, Supplementary Tables S3 and S4). Corresponding SusD-like proteins were detected in all years, but most strongly throughout the blooms of 2009 and 2011. Their protein identities to the isolate PULs ranged from 58–59% (Supplementary Table S4).

#### Sulfated β-xylan

One SusC and two SusD-like proteins likely targeting a sulfated β-xylan were expressed in the mid and late stages of the phytoplankton bloom of 2009, peaking at 0.08 %NSAF for SusC-like proteins and 0.07 %NSAF for SusD-like proteins. Their identities to homologs of the sulfated β-xylan PUL of *Formosa* sp. Hel3_A1_48 (Fig. 5b, PUL136, Supplementary Table S3) was 53% and 49–53%, respectively (Supplementary Table S4).

#### Beta-mannan

Homologs with high identities to SusC/D-like proteins occurring in a predicted β-mannan PUL from *Muricauda* sp. MAR_2010_75 were strongly expressed during blooms from 2009 to 2011, peaking on April 28th 2011 for SusC (0.22 %NSAF) and SusD (0.25 %NSAF) homologs. No SusC-like proteins of putative β-mannan PULs were detected in 2012 and SusD-like expression was likewise much weaker. The expressed SusC-like proteins were 52–60% identical to the ones from the β-mannan PUL of *Muricauda* sp. MAR_2010_75 (PUL266, Supplementary Table S3) and the SusD-like proteins showed 45–51% identity (Supplementary Table S4). The predicted β-mannan PUL of *Muricauda* sp. MAR_2010_75 harbors two pairs of SusC/D-like proteins. The one with expressed in situ homologs did not cluster with those from other beta-mannan PULs in our SusC/D trees. Hence the two SusC/D-like pairs might target different oligosaccharides. As some PULs can be induced by substrates other than those that they degrade, it is possible that the substrate that led to the upregulation of the in situ homologs was not a beta-mannan. Proteomic studies of this PUL in *Muricauda* sp. MAR_2010_75 are required to clarify regulation of this PUL and to interpret the in situ data.

#### Arabinan

SusD-like proteins potentially targeting an arabinan were expressed at late phytoplankton blooms stages during all four years with at least 0.05 %NSAF. However, their identities to the SusD-like protein of a predicted arabinan PUL from *Muricauda* sp. MAR_2010_75 were only 26–30% (Supplementary Figure S2I, PUL267, Supplementary Tables S3 and S4).

In summary, comparative analyses of SusC/D homolog expression are indicative of a successive utilization of different polysaccharides over the course of phytoplankton blooms. This agrees with successive changes in the microbial community composition during bloom events that we reported earlier on [32, 36].

## Discussion

PUL function predictions in this study are based on sequence similarity analyses and thus cannot rival time-consuming laboratory-based functional studies in terms of accuracy. Knowledge on polysaccharides from marine algae, in particular from microalgae, is still sparse and thus false predictions are possible. Still, the holistic approach to analyze the PUL spectrum of a large number of isolates from a single habitat allows identification of recurrent and thus important PULs as targets for future functional studies and to build testable hypotheses on possible substrates.

We observed diverse polysaccharide degradation capacities among North Sea *Flavobacteriia* with no distinct correlation to taxonomy. Even isolates from identical genera often featured notably diverging PUL repertoires and genome sizes (e.g., *Polaribacter*, *Maribacter,* and *Cellulophaga*), substantiating earlier data [14]. Our findings suggest that a species’ PUL repertoire is more dependent on its distinct ecological niche, whereas its phylogeny is of secondary importance. This corroborates the hypothesis that PULs are exchanged between *Flavobacteriia* through horizontal gene transfer [10].

The isolates’ PUL repertoires showcase that abundant, structurally simple substrates such as laminarin, α-1,4-glucans, and alginate are targeted by likewise conserved and frequent PULs. These substrates are likely so common that preserving the respective catabolic machinery is favorable for many marine *Flavobacteriia*. Diatom-derived chrysolaminarin has been estimated to amount to 5–15 petagrams of organic carbon annually [78] and accordingly laminarin-specific PULs were frequent in our surface water isolates. The four predicted laminarin PUL variants we identified might indicate that different laminarin types [49] are targeted by different PULs or that some of these PULs act as helper modules in laminarin degradation, as many species feature more than one laminarin PUL type (e.g., *Formosa* spp. Hel1_33_131 and Hel3_A1_48, *Gramella* sp. MAR_2010_102). Variant B contains predicted endo- and exo-acting β-1,3-glucan hydrolases (GH17) highly specific to laminarin degradation [45]. Variants A, C, and D only contain GH16 endo-1,3(4)-β-glucanases and may not be restricted to laminarin, but are potentially capable of degrading further mixed-linkage β-1,3/1,4-glucans, as recently shown for a similar conserved PUL in human gut *Bacteroidetes* [44]. Clustering of the SusC/D sequences of variants A, B and D in the SusC/D trees support that they bind the same substrate (Supplementary Figure S1A, B). Those of variant C, however, are located elsewhere, indicating that this PUL might indeed have an alternate function. Functional studies on model strains containing variant C (e.g., *Formosa* sp. Hel3_A1_48) and D (e.g., *Gramella* sp. MAR_2010_102) will be necessary to ultimately elucidate the functions of these PULs.

Alginate and α-1,4-glucan degradation capacities were prevalent in the isolates obtained from the > 20 µm retentate, which might be microalgae-associated, but were also common in many seawater isolates. Overall, laminarin, α-1,4-glucan, and alginate PULs are fairly conserved and make up over a quarter of all PULs in the isolates (115/400), suggesting that these are abundant polysaccharide substrates in North Sea coastal habitats that many microbes can consume and likely compete for.

Other substrates are utilized by fewer isolates, which implies that algal polysaccharide degradation is usually carried out by multiple resource-partitioning bacterioplankton species. In putative microalgae-associated isolates, these substrates include new FCSP variants, and xylose- and rhamnose-rich polysaccharides. Among surface water isolates, these substrates are sulfated α-mannans (likely an α-glucuronomannan glycoprotein), β-mannans, sulfated α-galactans and β-xylans, chitin, and (trehalose-like) α-1,1-glucans.

A major result of this study is the substrate-specific clustering of both SusC- and SusD-like proteins. The strong tendencies of SusC and SusD homologs to occur in corresponding substrate-specific clusters in both trees, resulting in similar tree topologies, suggest coevolution of these two proteins. This hypothesis is corroborated by recent X-ray crystallography findings showing complex formation of two SusC- and SusD-like proteins of *B. thetaiotaomicron* [7]. Clustering was more pronounced for structurally conserved, simple polysaccharides than for the heterogeneous and partially new substrates described in this study. This is expected, as heterogeneous substrates are attacked at multiple points resulting in a variety of structurally different oligosaccharides for uptake. Furthermore, broad substrate classes that currently can only be defined as, e.g., FCSPs or xylose-containing substrates might actually represent multiple chemically rather different substrates. Hence, improvement of functional clustering is to be expected once more detailed knowledge on algal polysaccharides structures is available.

We here provide first metaproteomic data indicating that high-resolution expression analysis of SusC/D homologs may be used for monitoring changes in microbial polysaccharide degradation activity. This provides a proxy on which polysaccharides are important at a given time and space in marine carbon cycling. Considering our still incomplete knowledge, only expressed SusC/D homologs exhibiting a high level of sequence identity to functionally annotated or characterized SusC/D sequences should be considered. Absence of such expressed homologs, however, does not preclude that a respective substrate may be targeted by an as yet unknown SusC/D system. This current limitation notwithstanding, our approach provides a new method to identify environmentally relevant polysaccharide substrates that due to their structural complexity are still difficult to identify by direct chemical analysis.

## Electronic supplementary material

Supplementary Text

Supplementary Figure S1A

Supplementary Figure S1B

Supplementary Figure S2

Supplementary Table S1

Supplementary Table S2

Supplementary Table S3

Supplementary Table S4

Supplementary Table S5
