# Supplementary material for: Polysaccharide utilization loci of North Sea Flavobacteriia as basis for using SusC/D-protein expression for predicting major phytoplankton glycans
Source: ISME J. 2018 Aug 15;13(1):76–91. doi: 10.1038/s41396-018-0242-6 (PMC6298971; doi:10.1038/s41396-018-0242-6)
Supplement: Supplementary file 1 — Supplementary Text [file 41396_2018_242_MOESM1_ESM.docx]

***The ISME Journal - Supplementary Text***

**Polysaccharide Utilization Loci of North Sea *Flavobacteriia* as Basis for Using SusCD-Protein Expression for Predicting Major Phytoplankton Glycans**

Lennart Kappelmann^1^, Karen Krüger^1^, Jan-Hendrik Hehemann^1,2^, Jens Harder^1^, Stephanie Markert^3,4^, Frank Unfried^3,4^, Dörte Becher^5^, Nicole Shapiro^6^, Thomas Schweder^3,4*^, Rudolf I. Amann^1*^, Hanno Teeling^1*^

^1^ Max Planck Institute for Marine Microbiology, Bremen, Germany

^2^ Zentrum für Marine Umweltwissenschaften, Bremen, Germany

^3^ Pharmaceutical Biotechnology, University Greifswald, Greifswald, Germany

^4^ Institute of Marine Biotechnology, Greifswald, Germany

^5^ Institute for Microbiology, University Greifswald, Greifswald, Germany

^6^ DOE Joint Genome Institute, Walnut Creek, CA, USA

^*^ Corresponding authors:

Hanno Teeling, Max Planck Institute for Marine Microbiology, Celsiusstraße 1, 28359 Bremen, e-mail: [hteeling@mpi-bremen.de](mailto:hteeling@mpi-bremen.de), phone: +49 421 2028 976

Thomas Schweder, University Greifswald, Pharmaceutical Biotechnology, Friedrich-Ludwig-Jahn-Str. 17, 17487 Greifswald, e-mail: schweder@uni-greifswald.de, phone: +49 3834 420 4212

Rudolf I. Amann, Max Planck Institute for Marine Microbiology, Celsiusstraße 1, 28359 Bremen, e-mail: [ramann@mpi-bremen.de](mailto:ramann@mpi-bremen.de), phone: +49 421 2028 930

**Running title:** PULs in North Sea *Flavobacteriia*

**This Supplementary Information PDF file contains:**

- Supplementary Results and Discussion with references

Supplementary Tables are available as separate Excel files.

**Supplementary Results**

*Absence of susCD-like genes in PULs*

In most PULs, the *susCD*-like gene pair was present and served as a major identifier along with the co-occurring CAZymes. Constant exceptions were the putative digeneaside (α-D-mannopyranosyl-(1→2)-D-glycerate) PULs, which did not contain *susD* homologs.

The *susCD*-like gene pair was also sometimes absent, e.g. in laminarin PUL variant B of (Figure 2B). While most of these strains possessed another laminarin PUL for laminarin oligosaccharide import, certain organisms did not. In that case, the *susCD*-like gene pair could sometimes be found completely isolated elsewhere in the genome (e.g. in *Nonlabens* sp. Hel1_33_55, laminarin PUL 276; corresponding *susCD* pair locus tag Ga0052909_2263 & _2264, displaying 56% (*susC*-like) and 68% (*susD*-like) amino acid identity to laminarin PUL associated *susCD*-like genes of the laminarin PUL 186 of *Leeuwenhoekiella* sp. MAR_2009_132, locus tag P162DRAFT_0226 & _0227). These results support recent findings that polysaccharide utilization is not necessarily encoded in one canonical locus, and should therefore not be as narrowly defined as originally suggested (1–3).

*Transcriptional regulators and Por secretion system genes* *in the isolate PULs*

Additional regularly occurring genes in marine *Flavobacteriia* PULs code for inner membrane sensor-regulator systems that dictate the activity of PUL genes when a substrate becomes available (4). These include substrate-specific transcriptional regulator family genes such as *luxR* in β-glucan PULs (prominently laminarin), *lacI* in α-glucan PULs and *gntR* in PULs targeting alginate and certain rhamnose-containing substrates. Other prevalent PUL-associated regulators were AraC-type DNA-binding domain-containing proteins and extra-cytoplasmic function (ECF) sigma/anti-sigma factors. AraC-type DNA-binding domain-containing proteins were often co-located with two-component regulator propellers and other uncharacterized membrane proteins. They were characteristically found in PULs targeting larger, sulfated substrates rich in fucose (FCSP), rhamnose, xylose and carrageenan, as well as unsulfated β-mannans. Transcriptional regulators of this family belong to hybrid two-component systems (HTCS), which contain the DNA-binding response regulator and a transmembrane sensor histidine kinase as a single peptide fused to a carbohydrate-sensing domain in the periplasm (5). ECF sigma factors were prominently found in PULs targeting sulfated rhamnans and xylose- and NAG-containing substrates.

Lastly, many PULs featured genes containing predicted Por secretion system C-terminal sorting domains. In the soil and freshwater bacterium *Flavobacterium johnsoniae*, the Por secretion system was found to be involved in the secretion of proteins for the assembly of the gliding motility apparatus and, interestingly, the secretion of chitinases (6), indicating that genes containing these domains could potentially be involved in the secretion of CAZymes to the environment for the initial endo-cleaving activity in the polysaccharide degradation cascade.

*Substrate specificities*

*Sulfated α-rhamnose-containing substrates*

In seven isolates we detected eleven PULs likely targeting sulfated α-rhamnose-containing substrates. These PULs feature predicted GH78 α-L-rhamnosidases and were often complemented by GH106 family α-L-rhamnosidases (Supplementary Figure S2A). Sulfated rhamnans have been reported for green macroalgae (7). Some of these rhamnose-PULs additionally contained predicted GH105 family rhamnogalacturonyl hydrolases, which cleave rhamnose from uronic acids. As GH78 family enzymes have also been shown to act on rhamnogalacturonans, it seems likely that these GH105 complemented rhamnose-PULs target rhamnogalacturonans, e.g. the matrix polysaccharide ulvan in green macroalgae (8).

*Sulfated α-galactose-containing substrates*

Four PULs with characteristic GH110 family α-galactosidases were rich in sulfatases and likely target sulfated, galactose-rich substrates. Sulfated galactans have been described in red macroalgae and seagrass (9). Two of those PULs additionally featured GH82 family carrageenases and predicted GH2 family β-galactosidases (Supplementary Figure S2B), indicating degradation of the red macroalgal cell wall constituent carrageenan that consists of D-galactose-4-sulfate and 3,6-anhydro-d-galactose-2-sulfate units bound by alternating α-1,3 and β-1,4 linkages (10). A different carrageenan is likely targeted in three different sulfatase-rich PULs (Supplementary Figure S2C; PULs 43, 51 of *Cellulophaga* spp. RHA19, _52, PUL 361 of *Zobellia amurskyensis* MAR_2009_138) that possess a combination of CAZymes distantly related to families GH127 and GH129 and a GH2 β-galactosidase. A similar, carrageenan-specific PUL, which lacks the GH2 and *susCD*-like genes in the locus itself, was recently characterized in *Zobellia galactanivorans* DsiJ^T^ (2). The GH127 and GH129 proteins encoded in that PUL showed exo-lytic α-1,3-(3,6-anhydro)-D-galactosidase activity on carrageenan oligosaccharides, hence displaying novel enzymatic activities for the two CAZyme families.

*Pectin*

GH105 family CAZymes also frequently occur in another type of PUL, which is equipped with a rich set of CAZymes suggesting degradation of pectin. 14 PULs were identified in ten isolates (Supplementary Table S3), which characteristically feature GH28 α-1,4-polygalacturonases and GH88 unsaturated β-glucuronyl hydrolases, pectate lyases of the families PL1, PL9 and PL10 and carbohydrate esterase of the families CE8 and CE12 (Supplementary Figure S2D). CE8 are pectin methylesterases and CE12 family enzymes have been described as pectin acetylesterases, rhamnogalacturonan acetylesterases and acetyl xylan esterases. All CAZymes observed in this type of PUL were identified in fungal degradation of pectins (11). Barbeyron and colleagues found a PUL with similar pectin-specific CAZymes (PL1 and GH28) in the marine flavobacterium *Z. galactanivorans* DsiJ^T^, yet no pectin degradation could be shown in culture. They hypothesized that these enzymes could therefore display new, uncharacterized specificities towards an as yet undescribed, pectin-like marine substrate.

*Further potential substrates: N-acetylglucosamine and chitin, digeneaside, fructose and trehalose*

The flavobacterial isolates featured plenty of additional PULs (Supplementary Table S3). Eleven isolates can assimilate digeneaside (Supplementary Figure S2E), which occurs in exudates of red macroalgae (12), through a PUL-associated GH63 family mannosylglycerate hydrolase and a co-occurring glycerate kinase, which convert digeneaside into D-mannose 6-phosphate and 3-phospho-D-glycerate (13). This PUL does not encode a SusD-like protein and the TBDT is not annotated as SusC-like.

Ten isolate genomes contained PULs targeting β-N-acetylglucosamine (GlcNAc) through GH20 family β-hexosaminidases (Supplementary Figure S2F). GlcNAc is the monomeric unit of chitin, which is targeted by another set of CAZymes, GH18 and GH19 family chitinases (Supplementary Figure S2G). Both these CAZymes frequently possess chitin-binding CBM5 and CBM12 domains. Chitin targeting loci were found in six isolates although interestingly, two of those, belonging to the genus *Tenacibaculum*, did not contain an adjacent *susCD*-like gene pair. Both isolates, however, feature a GlcNAc-targeting (GH20) PUL. In fact, for *Tenacibaculum* sp. MAR_2010_89, it is the only PUL with a dedicated *susCD*-like gene pair. This indicates a GlcNAc-containing substrate, potentially chitin, as a polysaccharide of major importance to the *Tenacibaculum* clade. Chitin is found in the exoskeletons of crustaceans, but was also detected in crystalline fibers excreted by diatoms (14) and in the silica frustule of *Thalassiosira pseudonana* (15).

A fructose-containing substrate is targeted by a conserved PUL in six isolates featuring a GH32 β-fructosidase and a sugar kinase, likely a fructokinase (Supplementary Figure S2H). Five isolates showed capacities to tackle diverse sialic acid oligosaccharides through PUL-associated GH33 family sialidases (Supplementary Table S3).

Another PUL featuring a predicted GH127 gene framed by two GH51 genes was found in *Muricauda* sp. MAR2010_75 (Supplementary Figure S2I). The GH127 family has known β-L-arabinofuranosidase and 3,6-anhydro-D-galacosidase functions (2), while the GH51 family comprises mostly (but not exclusively) α-L-arabinofuranosidases. Considering presence of a L-arbabinose isomerase gene in this PUL, a mixed α- and β-linked, unsulfated arabinan is a likely substrate. However, a carrageenan would also be a possible target. Finally, an α-1,1-linked glucan, likely trehalose, is predicted to be targeted by two isolates that possess PULs containing a GH37 family α,α-trehalase and a GH13 family α-glucosidase (Supplementary Figure S2J).

**References**

1. Hemsworth GR, Déjean G, Davies GJ, Brumer H. Learning from microbial strategies for polysaccharide degradation. Biochem Soc Trans. 2016;44:94-108.

2. Ficko-Blean E, Préchoux A, Thomas F et al. Carrageenan catabolism is encoded by a complex regulon in marine heterotrophic bacteria. Nat Commun. 2017;8:1685.

3. Grondin JM, Tamura K, Déjean G, Abbott DW, Brumer H. Polysaccharide utilization loci: fueling microbial communities. J Bacteriol. 2017;199:e00860-16.

4. Martens EC, Lowe EC, Chiang H et al. Recognition and degradation of plant cell wall polysaccharides by two human gut symbionts. PLoS Biol. 2011;9:e1001221.

5. Ravcheev DA, Godzik A, Osterman AL, Rodionov DA. Polysaccharides utilization in human gut bacterium *Bacteroides thetaiotaomicron*: comparative genomics reconstruction of metabolic and regulatory networks. BMC Genomics. 2013;14:873.

6. McBride MJ, Zhu Y. Gliding motility and Por secretion system genes are widespread among members of the phylum *Bacteroidetes*. J Bacteriol. 2013;195:270-278.

7. Wang L, Wang X, Wu H, Liu R. Overview on biological activities and molecular characteristics of sulfated polysaccharides from marine green algae in recent years. Mar Drugs. 2014;12:4984-5020.

8. Lahaye M, Robic A. Structure and functional properties of ulvan, a polysaccharide from green seaweeds. Biomacromolecules. 2007;8:1765-1774.

9. Aquino RS, Landeira-Fernandez AM, Valente AP, Andrade LR, Mourão PA. Occurrence of sulfated galactans in marine angiosperms: evolutionary implications. Glycobiology. 2005;15:11-20.

10. Michel G, Chantalat L, Fanchon E, Henrissat B, Kloareg B, Dideberg O. The iota-carrageenase of *Alteromonas fortis*. A beta-helix fold-containing enzyme for the degradation of a highly polyanionic polysaccharide. J Biol Chem. 2001;276:40202-40209.

11. Benoit I, Culleton H, Zhou M et al. Closely related fungi employ diverse enzymatic strategies to degrade plant biomass. Biotechnol Biofuels. 2015;8:107.

12. Kremer BP. Taxonomic implications of algal photoassimilate patterns. Br Phycol J. 1980;15:399-409.

13. Barbeyron T, Thomas F, Barbe V et al. Habitat and taxon as driving forces of carbohydrate catabolism in marine heterotrophic bacteria: example of the model algae-associated bacterium *Zobellia galactanivorans* Dsij. Environ Microbiol. 2016;18:4610-4627.

14. Blackwell J, Parker KD, Rudall KM. Chitin fibres of the diatoms *Thalassiosira fluviatilis* and *Cyclotella cryptica*. J Mol Biol. 1967;28:383-385.

15. Tesson B, Masse S, Laurent G et al. Contribution of multi-nuclear solid state NMR to the characterization of the *Thalassiosira pseudonana* diatom cell wall. Anal Bioanal Chem. 2008;390:1889-1898.
