# Supplementary material for: Polysaccharide utilization loci of North Sea Flavobacteriia as basis for using SusC/D-protein expression for predicting major phytoplankton glycans
Source: ISME J. 2018 Aug 15;13(1):76–91. doi: 10.1038/s41396-018-0242-6 (PMC6298971; doi:10.1038/s41396-018-0242-6)
Supplement: Supplementary file 2 — Supplementary Figure S1A [file 41396_2018_242_MOESM2_ESM.pdf]

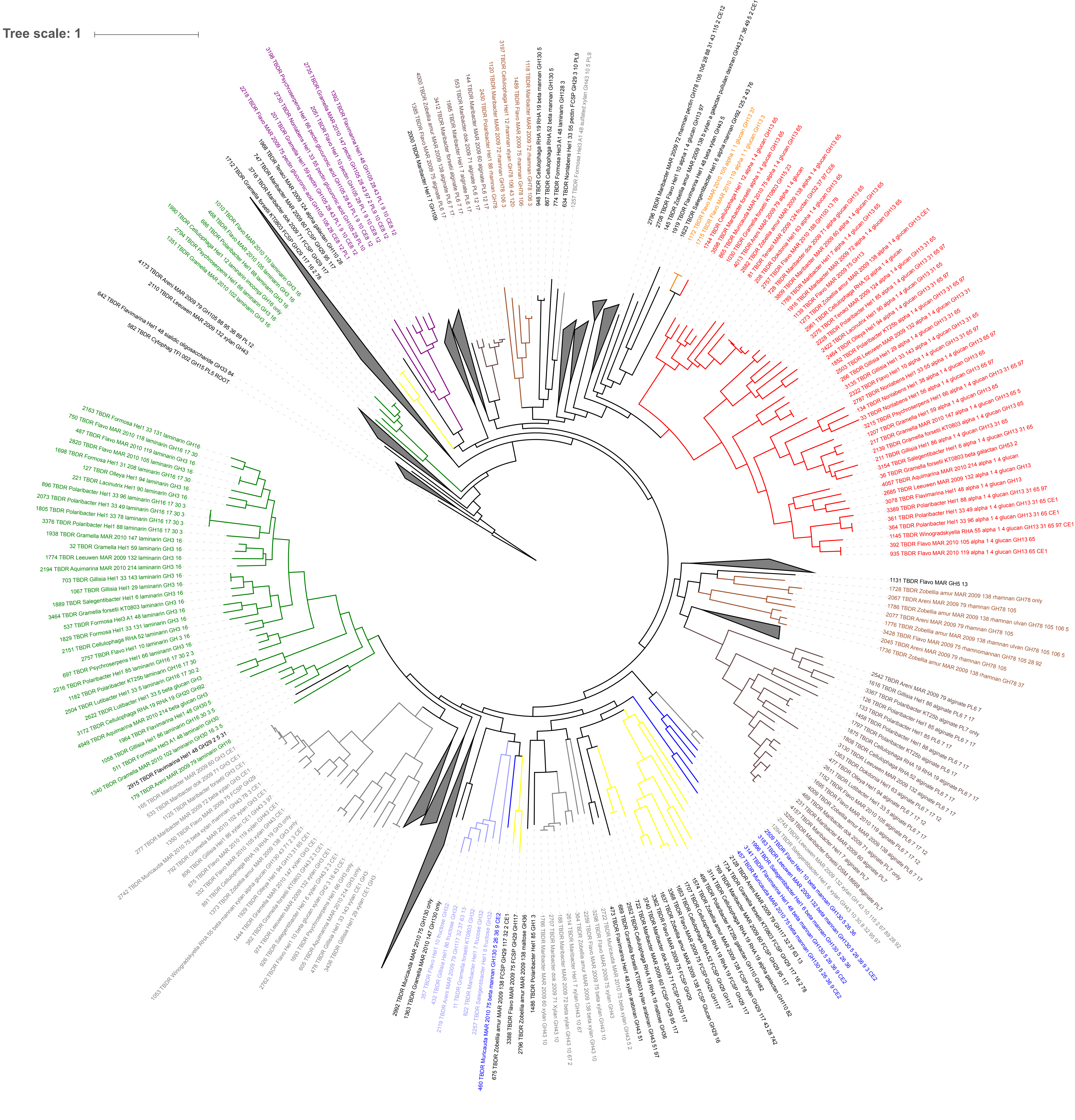

Supplementary Figure S1A. SusC-like protein tree showing functional, substrate-specific clustering. Protein sequences were aligned using the MAFFT v7.017 G-INS-i algorithm and trees were calculated using FastTree 2.1.5 approximate-maximum likelihood. Substrate predictions are depicted in colors. Labels indicate sequence identifier, isolate name, predicted substrate and PUL-associated CAZymes. The leading numbers of the sequences' labels corresponds to their GenDB region_IDs. Table S3 provides a conversion between these identifiers and the corresponding locus_tags in the published genomes.
