# Supplementary material for: Polysaccharide utilization loci of North Sea Flavobacteriia as basis for using SusC/D-protein expression for predicting major phytoplankton glycans
Source: ISME J. 2018 Aug 15;13(1):76–91. doi: 10.1038/s41396-018-0242-6 (PMC6298971; doi:10.1038/s41396-018-0242-6)
Supplement: Supplementary file 4 — Supplementary Figure S2 [file 41396_2018_242_MOESM4_ESM.pdf]

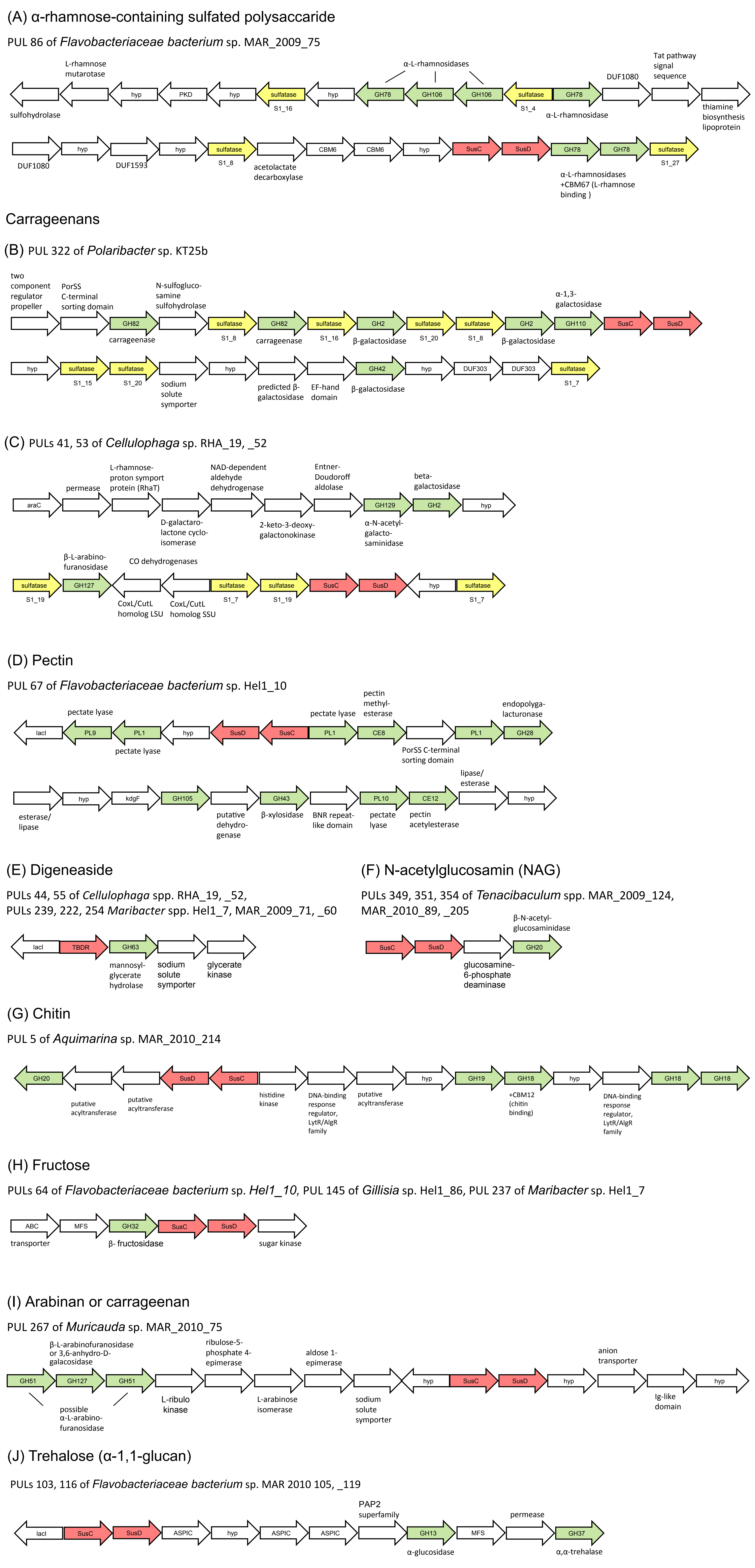

Supplementary Figure S2. PULs predicted to target (A) a sulfated, rhamnose-rich substrate, (B, C) carrageenans, (D) pectin, (E) digeneaside, (F) N-acetylglucosamine, (G) chitin, (H) fructose, (I) arabinan and (J) trehalose. For sulfatases, families and sub-families are indicated below the genes.
