# Supplementary material for: Polysaccharide utilization loci of North Sea Flavobacteriia as basis for using SusC/D-protein expression for predicting major phytoplankton glycans
Source: ISME J. 2018 Aug 15;13(1):76–91. doi: 10.1038/s41396-018-0242-6 (PMC6298971; doi:10.1038/s41396-018-0242-6)
Supplement: Supplementary file 5 — Supplementary Table S1 [file 41396_2018_242_MOESM5_ESM.pdf]

	Organism	Isolation source	Cultivation medium	Genome size [Mbp]	16S rRNA operons	No. of PULs	Sulfa-tases	CAZymes	Degrad. CAZYmes (GH, PL, CE)	Pepti-dases	Peptidase: CAZyme ratio
	Polaribacter sp. Hel1_88	seawater	HaHa agar	4.0	3	7	26	127	70	167	1.31
	Polaribacter sp. KT25b	seawater	ASM	4.0	3	11	40	146	90	142	0.97
	Polaribacter sp. Hel1_85	seawater	HaHa agar	3.9	4	8	31	139	73	162	1.17
	Polaribacter sp. Hel1_33_49	seawater	liquid Haha	3.0	3	5	12	105	50	136	1.30
	Polaribacter sp. Hel1_33_78	seawater	liquid Haha	3.2	3	6	7	107	51	149	1.39
	Polaribacter sp. Hel1_33_96	seawater	liquid Haha	3.1	4	4	7	104	50	135	1.30
	Tenacibaculum sp. MAR_2009_124	particle net	NAG	5.5	6	7	8	117	55	226	1.93
	Tenacibaculum sp. MAR_2010_89	porewater	HaHa agar	4.2	7	2	1	84	26	200	2.38
	Tenacibaculum sp. MAR_2010_205	seawater	HaHa agar	3.2	6	1	1	47	15	128	2.72
	Lutibacter sp. Hel1_33_5	seawater	liquid Haha	3.1	2	4	4	97	38	131	1.35
	Flavobacteriaceae bacterium sp. Hel1_10	seawater	HaHa agar	4.1	2	10	1	182	88	167	0.92
	Flavobacteriaceae bacterium sp. MAR_2010_10	porewater	HaHa agar	3.3	2	1	0	88	19	177	2.01
	Flavobacteriaceae bacterium sp. MAR_2010_118	porewater	HaHa agar	3.3	2	1	0	97	23	202	2.08
	Lacinutrix sp. Hel1_90	seawater	HaHa agar	3.8	3	4	1	109	35	169	1.55
	Olleya sp. Hel1_94	seawater	HaHa agar	3.6	6	5	0	112	37	145	1.29
	Winogradskyella sp. RHA_55	mussel surface	2216E	3.7	3	6	4	131	40	162	1.24
	Psychroserpens sp. Hel1_66	seawater	HaHa agar	3.8	2	6	2	146	48	173	1.18
	Flavobacteriaceae bacterium sp. MAR_2010_105	porewater	HaHa agar	3.3	2	11	4	133	64	158	1.19
	Flavobacteriaceae bacterium sp. MAR_2010_119	porewater	HaHa agar	3.2	2	9	2	119	50	162	1.36
	Flavobacteriaceae bacterium sp. MAR_2010_188	porewater	HaHa agar	3.8	2	6	1	148	49	196	1.32
	Formosa sp. Hel1_33_131	seawater	liquid Haha	2.7	2	6	7	63	24	114	1.81
	Formosa sp. Hel3_A1_48	seawater	liquid Haha	2.0	2	7	16	66	33	91	1.38
	Formosa sp. Hel1_31_208	seawater	liquid Haha	3.1	2	1	0	92	25	181	1.97
	Dokdonia sp. Hel1_5	seawater	HaHa agar	3.6	3	0	0	98	19	182	1.86
	Dokdonia sp. Hel1_53	seawater	HaHa agar	2.9	4	0	0	89	22	140	1.57
	Dokdonia sp. Hel1_63	seawater	HaHa agar	3.5	6	2	0	103	28	156	1.51
	Aquimarina sp. MAR_2010_214	seawater	HaHa agar	6.0	3	9	0	133	55	269	2.02
**dominated by 20 µm particle net isolates**	*Muricauda* sp. MAR_2010_75	seawater	HaHa agar	4.4	2	10	5	140	72	194	1.39
	Flavobacteriaceae bacterium sp. MAR_2009_75	particle net	SYL	4.8	2	22	44	189	112	186	0.98
	Zobellia amurskyensis MAR_2009_138	particle net	cellobiose	5.4	3	40	95	236	154	181	0.77
	Maribacter forsetii DSM_18668	seawater	2216E	4.5	3	9	12	118	59	195	1.65
	*Maribacter dokdonensi*s MAR_2009_71	particle net	NAG	4.6	3	9	28	145	77	207	1.43
	Maribacter dokdonensis MAR_2009_60	particle net	NAG	4.5	3	8	20	146	74	189	1.29
	Maribacter sp. MAR_2009_72	particle net	glucose	4.3	3	9	14	159	82	197	1.24
	Maribacter sp. Hel1_7	seawater	HaHa agar	4.8	4	12	8	144	72	204	1.42
	Arenibacter palladensis MAR_2009_79	particle net	glucose	5.4	3	25	60	189	117	186	0.98
	Cellulophaga sp. Hel1_12	seawater	HaHa agar	4.0	3	5	11	96	46	202	2.10
	Cellulophaga sp. RHA_19	*P. lanosa*	CAA	3.9	4	12	27	133	73	143	1.08
	Cellulophaga sp. RHA_52	particle net	2216E	3.7	4	9	14	119	62	145	1.22
	Ulvibacter sp. MAR_2010_11	porewater	HaHa agar	3.0	2	0	3	83	16	156	1.88
	Gramella forsetii KT0803	seawater	ASM	3.8	3	10	2	151	63	160	1.06
	Gramella sp. MAR_2010_102	porewater	HaHa agar	3.5	3	5	2	140	46	169	1.21
	Gramella sp. MAR_2010_147	porewater	HaHa agar	3.2	3	7	5	128	54	150	1.17
	Gramella sp. Hel1_59	seawater	HaHa agar	3.4	3	3	0	128	42	163	1.27
	Salegentibacter sp. Hel1_6	seawater	HaHa agar	4.2	3	9	1	178	81	167	0.94
	Gillisia sp. Hel1_29	seawater	HaHa agar	4.0	4	4	0	125	30	167	1.34
	Gillisia sp. Hel1_33_143	seawater	liquid Haha	3.5	5	3	1	119	31	157	1.32
	Gillisia sp. Hel1_86	seawater	HaHa agar	4.2	3	8	0	135	52	161	1.19
	Leeuwenhoekiella sp. MAR_2009_132	seawater	HaHa agar	4.3	4	17	6	205	117	160	0.78
	Flavimarina sp. Hel1_48	particle net	cellobiose	4.1	2	15	0	214	104	174	0.81
	Nonlabens sp. Hel1_56	seawater	HaHa agar	4.0	3	2	1	116	40	146	1.26
	Nonlabens sp. Hel1_38	seawater	HaHa agar	3.0	2	1	0	80	20	136	1.70
	Nonlabens sp. Hel1_33_55	seawater	liquid Haha	3.3	2	4	8	117	44	144	1.23
	min			2.02	2	0	0	47	15	91	0.77
	max			5.98	7	40	95	236	154	269	2.72
	**average**			**3.83**	**3.2**	**7.5**	**10.2**	**126.7**	**55.0**	**167.2**	**1.41**

Supplementary Table S1 Selected genomic information on the 53 North Sea *Flavobacteriia* of this study. Isolate order corresponds to the 16S rRNA phylogenetic tree (Figure 1).
